# Research on the mechanisms of natural products in radiation protection

**DOI:** 10.3389/fbioe.2025.1640682

**Published:** 2025-08-25

**Authors:** Xiaoxue Li, Luyao Yu, Hongchi Xu, Xiaowen Xing, Wenhui Wu, Yifei Feng, Li Ma, Zheng Zhou, Bailin Li, Ying He

**Affiliations:** ^1^ College of Food Science and Technology, Shanghai Ocean University, Shanghai, China; ^2^ Navy Special Medical Centre, Second Military Medical University, Shanghai, China

**Keywords:** ionizing radiation, radiation damage, natural products, preventive and therapeutic effect, mechanism

## Abstract

Radiation exposure initiates a cascade of reactions, including the release of reactive oxygen species, DNA double-strand breaks, and cellular apoptosis, leading to cell death, tissue damage, and potentially the development of cancer. Consequently, there is an urgent need to develop highly effective and low-toxicity radioprotective agents. Traditional chemically synthesized protective agents face significant limitations in clinical applicability due to their pronounced off-target toxicity, narrow therapeutic window, and high production costs. In recent years, bioactive natural compounds, including polysaccharides, polyphenols, saponins, alkaloids, and peptides, have emerged as key research targets for the next-generation of radioprotective drugs due to their low toxicity and multi-target synergistic effects. Notably, each class of compounds demonstrates distinct characteristics in its mechanisms of action. In comparison to synthetic drugs, these natural compounds exert protective effects primarily through three mechanisms: antioxidant activity, anti-apoptotic effects, and immune modulation. Additionally, they offer advantages such as abundant availability and high safety profiles. Current research must further elucidate the mechanisms of action of their active ingredients to establish a theoretical foundation for radiation protection in contexts involving radiation workers and potential nuclear emergencies. This article systematically elucidates the molecular mechanisms underlying radiation damage, summarizing the multidimensional protective effects and action pathways of natural products. Its objective is to provide both a theoretical foundation and technical insights for the development of novel radioprotectants.

## Highlight

1. Systematically summarize the multilevel damage mechanisms of ionizing radiation in biological systems, and illustrate the process using intuitive diagrams.
2. Comprehensively summarize recent advances in anti-radiation natural drugs and bioactive monomers, classifying them by structural characteristics.
3. Under the classification framework of five types of natural radiation-resistant components (polysaccharides, polyphenols, saponins, alkaloids, and polypeptides), the mechanism of action of the five types of natural radiation-resistant components (polysaccharides, polyphenols, saponins, alkaloids, and polypeptides) was analyzed in depth.
4. Combined with modern radiotherapy programs and cutting-edge research trends, the development strategy of a new generation of radiation protective agents based on natural products is proposed; By elucidating the targeting mechanism and action bias of various natural compounds, the transformation prospect of various natural compounds in the field of high-efficiency and low-toxicity radiation protection is demonstrated.

## 1 Introduction

Radiation, as a physical phenomenon of energy transfer, can be categorized into two main types based on its quantum energy threshold: ionizing radiation (IR) and non-ionizing radiation. Ionizing radiation can cause irreversible damage, such as DNA double-strand breaks and protein denaturation, through either direct ionization or the indirect generation of reactive oxygen species (ROS), as its photon energy exceeds 12.4 eV. The harmful effects of ionizing radiation are significantly greater than those associated with non-ionizing radiation, which primarily produces thermal effects. Research indicates that exposure to IR can cause damage at multiple levels: physically, it penetrates tissues and induces ionization events; chemically, it triggers the explosive generation of ROS; and biologically, it initiates cell cycle arrest and programmed cell death. Ultimately, this cascade of effects can lead to deterministic or stochastic outcomes, including acute radiation sickness and cancer.

Despite the considerable hazards associated with IR, its distinctive physical and chemical properties—including its penetration ability, controllable ionization capacity, and predictable interactions with matter—have facilitated its systematic application across several key sectors of modern society, such as healthcare, industry, and energy. In the medical field, X-rays and radioactive isotopes are extensively utilized for imaging diagnostics and cancer treatment, with computed tomography (CT) scans emerging as a standard method for disease screening. In industrial and agricultural applications, gamma rays are utilized for weld defect detection, food sterilization, and pest control, thereby significantly enhancing safety and efficiency in production. In the energy sector, ranging from nuclear power generation to the development of new materials, these innovative applications create numerous opportunities for humanity to advance clean energy. However, the inevitable radiation exposure associated with these application scenarios presents a persistent threat to human health.

In response to radiation damage, the development of protective agents has emerged as a crucial strategy. In contemporary clinical practice, chemically synthesized protective agents have become a standard intervention for radiation protection due to their capacity to mitigate radiation-induced oxidative damage and immune injury. This mitigation occurs through molecular mechanisms, such as scavenging free radicals, modulating immune cell activity, and effectively regulating the release of inflammatory mediators (Liu et al., 2023). For instance, the primary mechanism of the synthetic radioprotective agent Amifostine is centered on scavenging ROS that are generated during the initial stages of radiation exposure, thereby alleviating immediate oxidative stress damage (Parashar et al., 2025). Corticosteroids primarily alleviate symptoms by exerting potent anti-inflammatory and immunosuppressive effects, with their core mechanism involving the inhibition of key transcription factors, which subsequently reduces the production of pro-inflammatory cytokines (Batcik et al., 2025). However, the inherent challenge of a narrow therapeutic window associated with this class of drugs results in significant dose-dependent risks, which clinically manifest as long-term safety concerns, including hypotension during the acute phase, neurogenic vomiting, and central nervous system-induced drowsiness. These accompanying risks render these drugs a suboptimal choice within the framework of radiation protection treatment (Singh et al., 2025).

In recent years, natural products have attracted growing interest from researchers in drug development, owing to their benefits, including multi-target synergistic effects, low biological toxicity, and the vast diversity found in natural compound libraries (Nisar et al., 2022; Luo Z. et al., 2024). In comparison to synthetic drugs, natural products demonstrate superior biocompatibility and exhibit significant potential for the development of radioprotective agents, thereby becoming a focal point of research in the field of radiation medicine. However, translating the promising *in vitro* and animal model findings of natural radioprotectors into clinically viable therapeutics remains a significant challenge. Key obstacles include addressing poor bioavailability, optimizing complex pharmacokinetic profiles, and establishing robust clinical efficacy data for human application (Chen Y. et al., 2024). To bridge this translational gap between experimental and clinical research, recent advances have identified promising strategies to enhance the *in vivo* delivery and therapeutic efficacy of natural radioprotective compounds (Chen Y. et al., 2024), thereby providing critical pathways for realizing their untapped potential. Preliminary human studies demonstrate the clinical relevance of specific natural products in mitigating radiation-associated complications (Wang et al., 2024), yielding tangible benefits in specific clinical contexts. This systematic review elucidates the molecular mechanisms underlying IR damage, summarizes research progress on natural bioactive products with radioprotective effects, and focuses on analyzing their mechanisms of action. The validation of clinical utility necessitates the assessment of bioactivity in relevant human models, which is a crucial step in the process of clinical translation (Wang et al., 2022). However, current evidence primarily relies on long-term follow-up studies of radiation workers and individuals exposed to accidents, which lack proactive clinical trial translation of innovative drugs. In summary, this study establishes the theoretical and mechanistic foundation for the development of natural product-derived radioprotectants with enhanced translational potential and clinical applicability.

## 2 The mechanism of radiation damage to the organism

The mechanisms of IR damage to biological organisms involve both direct and indirect effects. The direct effect refers to the damage inflicted by IR on DNA and other cellular components. In contrast, the indirect effect arises from the generation of excessive ROS, which leads to oxidative stress and, consequently, causes cellular damage and cell death. Additionally, IR may activate inflammatory responses, impact the immune system, induce alterations in the cell cycle, and cause long-term genetic effects, resulting in various forms of damage to the organism (Talapko et al., 2024).

### 2.1 DNA damage

Cellular damage resulting from IR is one of the most prevalent biological effects, capable of disrupting the molecular structure of DNA within cells, leading to single-strand breaks and double-strand breaks. Simultaneously, IR can also induce DNA damage indirectly, primarily through the radiolytic decomposition of water and other organic molecules, which generates a substantial amount of ROS. These ROS attack DNA, leading to oxidative damage (Sun et al., 2025), which results in base damage and DNA-protein cross-links, among other consequences (Szatkowska and Krupa, 2020). It is important to note that oxidative damage to DNA caused by ROS can lead to the oxidation of purines and pyrimidines, as well as the formation of apurinic/apyrimidinic sites, resulting in single-strand breaks (Maleki et al., 2024).

If DNA damage is not repaired, it may result in genetic mutations, chromosomal abnormalities, and subsequently trigger cancer, neurodegenerative diseases, and hereditary disorders. Currently, DNA damage repair primarily occurs through two pathways: non-homologous end joining and homologous recombination. Non-homologous end joining primarily functions during the G1 phase and early S phase of the cell cycle, while homologous recombination is more active during the S phase and G2 phase (Yang et al., 2025).

**FIGURE 1:**
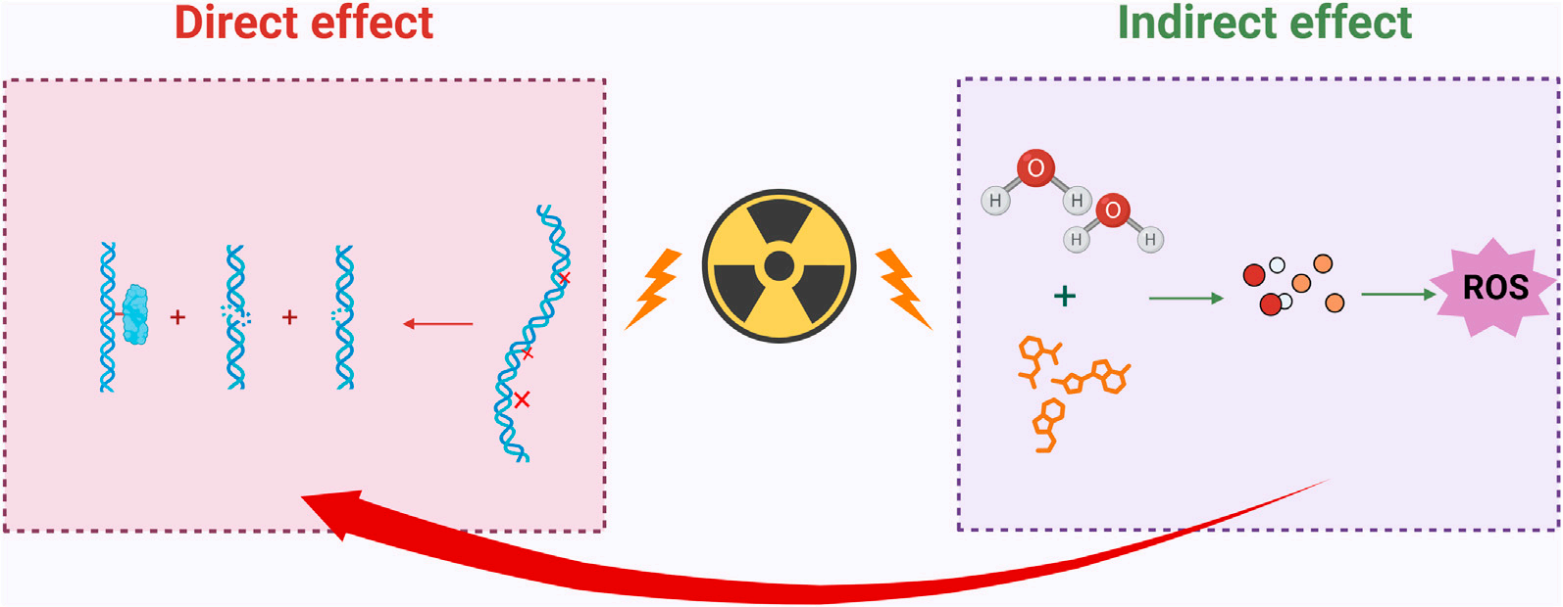
IR induces both direct and indirect DNA damage. Direct damage can lead to single-strand breaks, double-strand breaks, and DNA-protein crosslinks in DNA; indirect damage indirectly harms DNA by decomposing water molecules and other organic molecules, generating a large amount of ROS; Created in https://BioRender.com.

The DNA damage induced by IR is illustrated in Figure 1.

### 2.2 Causes oxidative stress

IR directly damages biomolecules through ionization or excitation; however, its primary threat to DNA and cellular function typically arises from a cascade of oxidative stress reactions (Chandimali et al., 2025). When high-energy radiation penetrates cells, its energy can ionize water molecules, leading to the generation of numerous ROS, including hydroxyl radicals, superoxide anions, and hydrogen peroxide. ROS exhibits strong oxidizing properties. Under normal conditions, a balance exists between the levels of free radicals and antioxidant defenses, which helps prevent cellular and tissue damage caused by reactive intermediates (Yahyapour et al., 2018). However, an excess of ROS can disrupt cellular redox balance, leading to damage to cellular structures and functions (Sharma et al., 2023; Mohamed et al., 2024).

#### 2.2.1 The impact of ROS on lipid metabolism

The regulation of lipid metabolism is essential for maintaining the body’s metabolic homeostasis. Disruption of lipid metabolism is linked to various diseases, including obesity, diabetes, cardiovascular diseases, and certain types of cancer (Wei et al., 2025). Research indicates that ROS can induce lipid peroxidation reactions, resulting in the production of harmful peroxidative products that subsequently damage the structure and function of cell membranes. Additionally, ROS can activate the nuclear factor kappa B (NF-κB) and the phosphatidylinositol 3-kinase/protein kinase B (PI3K/Akt) signaling pathways, which serve as key regulatory factors in the inflammatory response (Luo J. et al., 2024). Therefore, this activation not only promotes the release of inflammatory mediators but also interferes with insulin signaling, altering the utilization of glucose in adipose tissue. Ultimately, this results in a reduction of fatty acid release, further exacerbating disturbances in lipid metabolism (Li et al., 2025).

#### 2.2.2 The impact of ROS on mitochondria

Mitochondrial dysfunction is a critical pathological component in the mechanism of cellular damage associated with insulin resistance. As the central hub of cellular energy metabolism, the mitochondrial respiratory chain—particularly complexes I and III—undergoes electron leakage induced by insulin resistance, which directly leads to the excessive production of ROS, such as superoxide anions (O_2_^−^) (Zong et al., 2024). Due to the absence of histone protection and the proximity of mitochondrial DNA (mtDNA) to sites of ROS generation, the mutation rate of mtDNA can be 10 to 100 times higher than that of nuclear DNA. These mutations exacerbate the production of ROS by disrupting the assembly of respiratory chain complexes and impairing the function of ATP synthase, subsequently activating apoptotic signaling pathways. Clinical evidence suggests that the frequency of mtDNA deletion mutations in populations exposed to radiation is significantly positively correlated with both neurodegenerative diseases and metabolic syndrome.

Recent research has demonstrated that the insulin-like growth factor-1 (IGF-1) signaling pathway is essential for regulating insulin sensitivity, cardiovascular homeostasis, and the maintenance of mitochondrial function. The IGF-1 signaling pathway regulates mitochondrial morphology and function by participating in mitochondrial fission and cellular renewal processes, thereby influencing cellular tolerance to damage associated with insulin resistance. Research indicates that the IGF-1 signaling pathway enhances mitochondrial antioxidant capacity, reduces ROS generation, and promotes mitochondrial DNA repair by activating the downstream PI3K/Akt pathway (Aghdam et al., 2020). Furthermore, IGF-1 promotes the clearance of damaged mitochondria and the proliferation of healthy mitochondria by regulating mitochondrial dynamics, thereby restoring normal mitochondrial function. These research findings suggest that treatment strategies targeting IGF-1 as a central focus may serve as a powerful and effective therapy for addressing mitochondrial dysfunction caused by insulin resistance.

**FIGURE 2:**
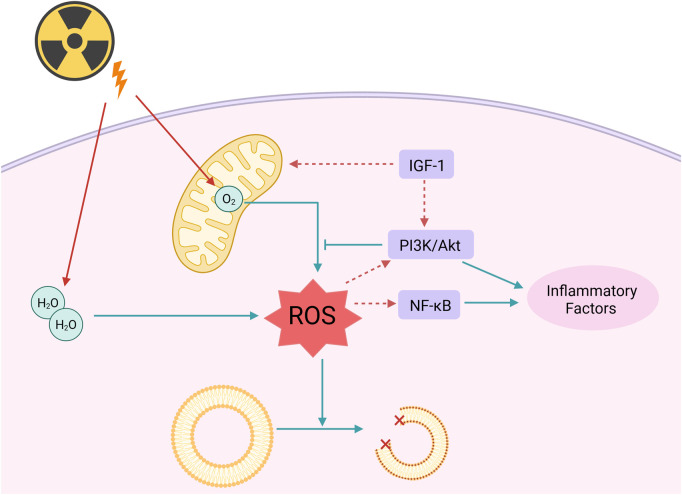
Radiation can directly or indirectly affect lipids and mitochondria through ROS. Radiation causes the decomposition of water molecules and oxygen produced by the mitochondrial respiratory chain into ROS, leading to lipid oxidation, and further activates the NF-κB and PI3K/Akt signaling pathways to generate inflammatory factors. IGF-1 can enhance the antioxidant capacity of mitochondria; Created in https://BioRender.com.

The mechanism of oxidative damage caused by IR to the organism is shown in Figure 2.

### 2.3 Inflammation

The inflammatory cascade induced by IR is a significant pathological mechanism contributing to tissue damage within the body. Research indicates that radiation sources, such as ionizing radiation (IR), can elicit both local and systemic inflammatory responses by inducing the release of pro-inflammatory cytokines, including NF-κB, tumor necrosis factor-alpha (TNF-α), interleukin-1 beta (IL-1β), and interleukin-6 (IL-6) (Sharapov et al., 2021; Sharma et al., 2023). Radiation-induced inflammatory responses occur not only through the classical inflammasome pathway but may also be exacerbated by non-classical pathways, including the release of extracellular vesicles and intercellular signaling (Gandhi and Chandna, 2017). Radiation primarily triggers the inflammatory response by activating the classical pathway of NF-κB. Radiation-induced DNA damage activates ataxia telangiectasia mutated (ATM) kinase, while ROS oxidatively modify IκB kinase beta (IKKβ). Additionally, damage-associated molecular patterns (DAMPs) interacting with Toll-like receptor 4 (TLR4) synergistically phosphorylate and degrade inhibitor of kappa B alpha (IκBα), thereby promoting the nuclear translocation of the p50/rela dimer and initiating the transcription of pro-inflammatory factors. This process is regulated by the ATM- IKK axis, the ROS-IKKβ interaction, and the TNF Receptor-Associated Factor 6 (TRAF6)-dependent signaling cascade. It is precisely balanced through the negative feedback mechanisms of IκBα resynthesis and A20 deubiquitination (Razani et al., 2011). This multidimensional activation pattern positions NF-κB as an “amplifier” within the radiation-induced inflammation network. In the ovarian radiation injury model, the upregulation of the Toll-Like Receptor 4 (TLR4)- NF-κB-TNF-α axis can create a self-reinforcing inflammatory microenvironment, thus exacerbating tissue dysfunction.

**FIGURE 3:**
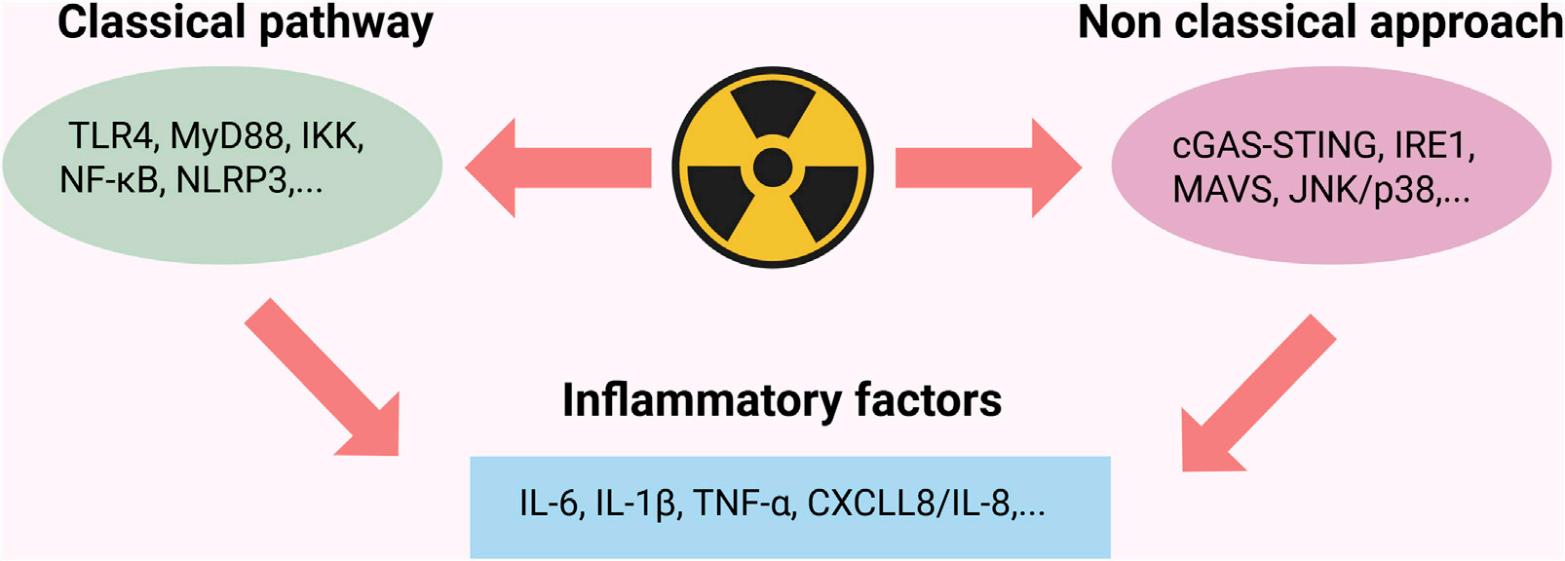
Radiation triggers inflammatory response through classical and non classical pathways. The classical pathway activates NF-κB via the TLR4/MyD88/IKK complex cascade, upregulating the expression of pro-inflammatory factors such as TNF-α and IL-6; the non-classical pathway promotes the secretion of IL-1β and CXCL8/IL-8 through the activation of the cGAS-STING pathway and endoplasmic reticulum stress sensing; Created in https://BioRender.com.

The mechanism of IR induced inflammatory response in the body is shown in Figure 3.

### 2.4 Apoptosis

Apoptosis plays a critical role in maintaining normal development, tissue homeostasis, and physiological functions in organisms. IR can disrupt the natural process of programmed cell death, which eliminates unnecessary cells in the body, resulting in the premature apoptosis of healthy cells. This phenomenon is primarily mediated through pathways, including the mitochondrial pathway (Cao et al., 2019), the endoplasmic reticulum pathway (Rahmani et al., 2023), and the death receptor pathway (Pistritto et al., 2016).

#### 2.4.1 Mitochondrial pathway

The mitochondrial pathway functions as the primary executor of programmed cell death and acts as a “signal integration hub” in radiation-induced apoptosis. Radiation disrupts mitochondrial redox homeostasis by either direct ionization or the indirect generation of ROS. On one hand, ROS target mitochondrial membrane phospholipids, resulting in lipid peroxidation and impairing the function of the respiratory chain complexes (Chen S. et al., 2024). Conversely, it stimulates the oligomerization of pro-apoptotic proteins Bax and Bak, leading to the formation of the mitochondrial outer membrane permeabilization (MOMP) pore, which facilitates the release of cytochrome c into the cytoplasm (Liu et al., 2025). Cytochrome c in the cytoplasm binds to Apoptotic Protease-Activating Factor 1 (Apaf-1), resulting in the formation of the apoptosome, which recruits and activates procaspase-9.

Cytochrome c in the cytoplasm binds to Apoptotic protease-activating factor 1 (Apaf-1) to form the apoptosome, which recruits and activates the precursor of caspase-9. This process subsequently cleaves the effector caspases-3 and -7, triggering terminal apoptotic events such as DNA fragmentation and nuclear membrane dissolution (Cao et al., 2019).

#### 2.4.2 Endoplasmic reticulum pathway

Endoplasmic reticulum (ER)-mediated apoptosis represents a crucial regulatory pathway of programmed cell death. Radiation can induce ER stress, which activates apoptosis signaling pathways associated with ER stress, ultimately resulting in cell apoptosis. Under physiological conditions, the ER maintains protein folding homeostasis through the unfolded protein response (UPR). Its core regulatory components—Protein Kinase RNA-like ER Kinase (PERK), Inositol-Requiring Enzyme 1α (IRE1α), and Activating Transcription Factor 6 (ATF6), coordinate signaling networks to respond to environmental stress (Rahmani et al., 2023). Radiation can elevate intracellular oxidative stress levels, disrupt ER homeostasis and function, lead to protein misfolding, and ultimately induce ER stress. If balance is not restored within a short period, the state of endoplasmic reticulum (ER) stress will activate a series of signaling pathways, including the PERK-eukaryotic translation initiation factor 2 alpha (eIF2α)-activating transcription factor 4 (ATF4)-C/EBP homologous protein (CHOP) pathway. CHOP is a pro-apoptotic protein whose increased expression induces apoptosis through various mechanisms, including the promotion of pro-apoptotic proteins such as Bax and the inhibition of the anti-apoptotic protein B-cell lymphoma 2 (Bcl-2), ultimately resulting in cell death (Abo-Zaid et al., 2023).

Recent studies have demonstrated that the novel anti-diabetic drug Ipragliflozin enhances glucose-induced insulin secretion, promotes β-cell proliferation, and mitigates β-cell apoptosis. Simultaneously, it mitigates stress-mediated β-cell apoptosis by regulating endoplasmic reticulum homeostasis, a process involving negative feedback from the CHOP/GADD34 pathway and signal transduction mediated by Activating Transcription Factor 3 (ATF3) or Stromal Cell-Derived Factor 2-Like 1 (SDF2L1) (Li et al., 2021).

#### 2.4.3 Death receptor pathway

The death receptor-mediated extrinsic apoptotic pathway serves as a crucial regulatory mechanism of programmed cell death, focusing on the specific recognition and signal transduction between death receptors on the cell membrane and their corresponding ligands. Radiation can activate death receptors on the cell surface, thereby inducing apoptosis. In the classical pathway involving Factor Associated Suicide/Fas ligand (Fas/FasL) signaling, the Fas receptor interacts with FasL, which is predominantly secreted by activated T cells, natural killer (NK) cells, and specific malignant tumor cells, via its extracellular domain. This interaction induces receptor trimerization and a conformational change, which subsequently exposes the intracellular death domain (DD). This domain interacts with the death domain (DD) region of the adaptor protein Fas-Associated Death Domain (FADD), thereby forming the primary framework of the death-inducing signaling complex (DISC). The maturation process of the DISC involves the recruitment and autocatalytic activation of the pro-caspase-8 precursor. Activated caspase-8 cleaves and activates the downstream effector caspases-3 and -7, ultimately triggering hallmark events of apoptosis, including chromatin condensation and DNA fragmentation (Newton et al., 2024).

In addition to the Fas system, the TNF-related apoptosis-inducing ligand (TRAIL) receptors and DR5 hold greater clinical significance in tumor-selective apoptosis. TRAIL activates a death-inducing signaling complex (DISC) assembly mechanism that is analogous to that of the Fas pathway by binding to the Death Receptor 4 (DR4) and Death Receptor 5 (DR5), which are highly expressed in tumor cells (Yuan et al., 2018). Owing to their low toxicity to normal tissues, over ten TRAIL receptor agonists have progressed to clinical trial stages. Research has demonstrated that certain tumors can evade TRAIL-induced apoptosis by upregulating decoy receptors DcR1 and DcR2 or the FLICE inhibitory protein (FLIP) (O'Leary et al., 2016).

**FIGURE 4:**
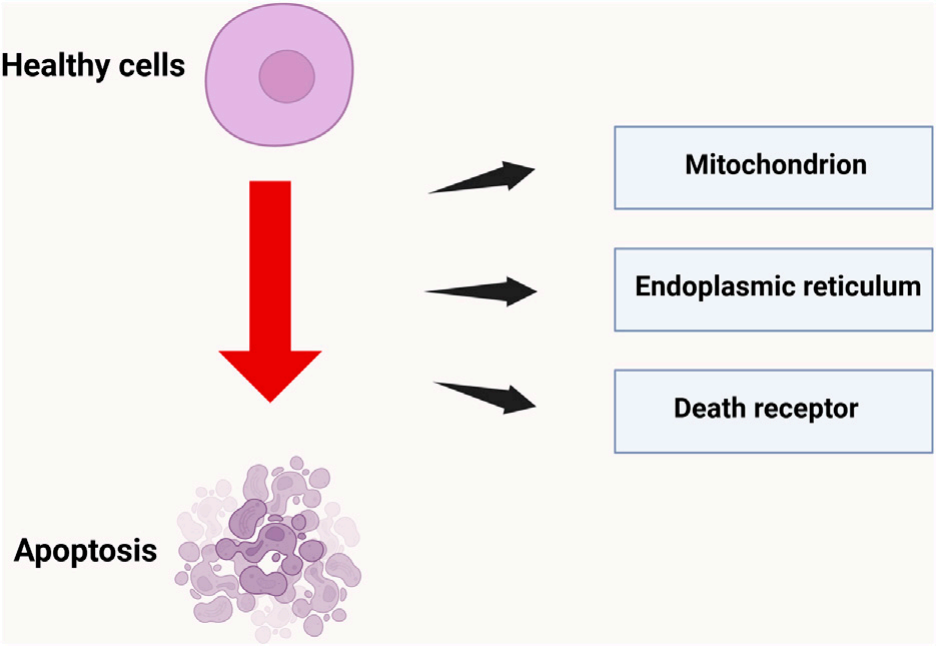
Three pathways of radiation-induced cell apoptosis. Radiation induces cell apoptosis through three core pathways: mitochondria, death receptors, and endoplasmic reticulum stress; Created in https://BioRender.com.

The mechanism of cell apoptosis induced by IR is shown in Figure 4.

## 3 Pathways of natural products in radiation protection

In recent years, the increasing demand for radiation protection, along with ongoing advancements in natural medicine research, has generated considerable interest in the application of natural products in the field of radiation protection. Active ingredients such as polyphenols, polysaccharides, alkaloids, saponins, and peptides, which are abundantly found in plants, animals, and microorganisms, can exert protective effects against radiation through synergistic mechanisms that involve multiple targets and pathways.

### 3.1 Polyphenols

Polyphenolic compounds, recognized as one of the most abundant families of plant secondary metabolites, exhibit substantial antioxidant activity. They are commonly found in natural resources, including tea, blueberries, and traditional Chinese herbal medicines. Characterized by multiple phenolic hydroxyl groups, these compounds exhibit strong free radical scavenging capabilities and redox regulation properties (Li et al., 2024), thereby demonstrating significant application value in the field of radiation damage protection. Recent studies have shown that polyphenols not only mitigate oxidative stress by directly neutralizing ROS generated by radiation but also establish a multi-layered radiation protection mechanism through the activation of DNA damage repair, inflammatory response, and cell death signaling pathways.

5,7-Dihydroxyflavone (5,7-DHF) is a naturally occurring plant flavonoid primarily derived from Oroxylum indicum, a member of the Bignoniaceae family. It serves as a raw material for the synthesis of pharmaceuticals aimed at treating cancer, reducing lipids, preventing cardiovascular and cerebrovascular diseases, as well as exhibiting antibacterial and anti-inflammatory properties. Research conducted by Mansour et al. demonstrated that significant apoptosis of entire brain cells occurs within 24 h in rats exposed to ionizing radiation (IR) at doses of ≤5 Gy. However, supplementation with 5,7-DHF effectively inhibits radiation-induced neuronal apoptosis. Mechanistic studies indicate that this protective effect is closely associated with the downregulation of caspase-3 activity (Mansour et al., 2017). Curcumin, a natural polyphenolic compound extracted from the rhizomes of Curcuma longa, has been shown to possess significant radioprotective properties. Studies have shown that when experimental rats were subjected to localized abdominal irradiation with a dose of 6 Gy of X-rays, their liver tissues exhibited typical pathological characteristics of radiation damage. Curcumin mitigated radiation-induced liver injury by inhibiting the NF-κB pathway, which reduced oxidative stress, inflammation, and apoptosis (Azmoonfar et al., 2025). Resveratrol (RES) is a naturally occurring polyphenolic compound. By establishing a mouse model of whole-body irradiation using 8 Gy of ^137^Cs-γ rays, Ali’s team found that RES pretreatment significantly inhibited radiation-induced inflammatory responses and cell apoptosis. Specifically, RES reduced the levels of inflammatory factors, including TNF-α, IL-1β, and interferon-gamma (IFN-γ), and decreased the expression of the proteins Bax and caspase-3 through the mitochondrial-dependent apoptotic pathway (Ali and Radwan, 2021). Hesperidin exhibits a variety of pharmacological effects and is primarily extracted from citrus fruits, tea, and olive oil. The research team led by Park discovered that it significantly alleviates intestinal barrier dysfunction induced by X-ray radiation by activating the Calmodulin-dependent Kinase Kinase/AMP-activated Protein Kinase (CaMKK/AMPK) pathway. This pathway regulates the expression of tight junction-related proteins, specifically occludin and claudin-4, thereby conferring a protective effect (Park and Yu, 2024). Moreover, polyphenols demonstrate potential synergistic effects. Research has shown that the combination of pterostilbene and silymarin with nicotinamide riboside and fibroblast stimulating lipoprotein one enables mice to survive lethal doses of γ-radiation. This is accomplished by decreasing the expression of the mutant p53 protein while increasing the expression of the pro-apoptotic Bax protein in breast cancer cells, thereby offering long-term protection to bodily tissues (Obrador et al., 2023). This innovative combination therapy approach opens entirely new avenues for the treatment of related diseases, demonstrating significant potential and application prospects. (−)-Epigallocatechin-3-gallate (EGCG) is a key active ingredient in green tea, exhibiting an exceptionally broad spectrum of biological activities and health benefits. Research conducted by Xie et al. has demonstrated that EGCG can enhance the survival rate of human intestinal epithelial cells (HIEC) following IR exposure, as well as reduce IR-induced DNA damage, apoptosis, and ferroptosis. The protective mechanisms include enhancing the nuclear translocation of Nuclear Factor Erythroid 2-Related Factor 2 (Nrf2), upregulating the expression of Heme Oxygenase-1 (HO-1) following IR exposure, and increasing the expression levels of Glutathione Peroxidase 4 (GPX4) and Solute Carrier Family 7 Member 11 (SLC7A11) after radiotherapy (Xie et al., 2020). Engelhardia roxburghiana Wall. leaves, a traditional medicinal plant utilized by the Zhuang ethnic group in Guangxi, exhibit unique pharmacological value and development potential. They are currently employed in the treatment of damp-heat diarrhea, hernia, and abdominal pain. The total flavonoids extracted from Engelhardia roxburghiana leaves (TFERL) demonstrate significant bioactivity. Recent research conducted by Wu’s team demonstrated that TFERL provides protection against IR-induced intestinal injury in mice by reducing levels of TNF-α and IL-6, while simultaneously enhancing the activity of superoxide dismutase (SOD) and glutathione (GSH). This modulation ultimately alleviates inflammation and oxidative stress triggered by IR (Wu et al., 2023). Naringenin (NAR), a dihydroflavonoid compound commonly found in citrus fruits, exhibits a range of biological activities, including antibacterial, anti-inflammatory, free radical scavenging, and antioxidant effects. It is extensively utilized in both the pharmaceutical and food industries. Research conducted by Ling’s team has demonstrated that NAR can mitigate radiation-induced intestinal damage by inhibiting the TRPV6 channel. This action reduces the activation of the apoptosis pathway induced by IR, subsequently downregulating the expression levels of cytochrome c oxidase subunit 4 (Cox-4), Bax, and cleaved caspase-3, while also decreasing the secretion of TNF-α, IL-1β, and IL-6 (Ling et al., 2024).Quercetin 3-rutinoside (Q-3-R) is primarily found in buckwheat, citrus fruit peels, and apples, and exhibits biological activities, including antioxidant and anti-inflammatory effects. Research conducted by Dutta et al. indicates that Q-3-R has protective potential against radiation-induced hematopoietic injury. Its mechanism of action involves the regulation of oxidative stress and apoptosis-related mediators in the spleen and bone marrow of mice. Specifically, pretreatment with Q-3-R effectively inhibits radiation-induced ROS production and restores serum IL-6 levels. Additionally, the study observed an upregulation of Bcl-2 expression, a downregulation of Bax expression, alterations in NF-κB activity, and a reduction in serum TNF-α levels. These differential regulatory phenomena collectively indicate that quercetin-3-rutinoside (Q-3-R) mediates its radioprotective effects by exerting anti-apoptotic and anti-inflammatory actions (Dutta et al., 2021). Urolithin A (UroA) is a naturally occurring polyphenol derivative that is metabolically produced by gut microbiota and belongs to the benzofuranone class of compounds. Research conducted by Zhang et al. has demonstrated that UroA can significantly reverse IR-induced dysbiosis of the intestinal microbiota in mice. Additionally, its protective mechanism may be linked to the inhibition of p53-mediated apoptosis as well as the remodeling of the intestinal microbiota (Zhang et al., 2021).

Polyphenols exhibit enhanced efficacy in scavenging radiation-induced free radicals, particularly the hydroxyl radical, and in inhibiting lipid peroxidation, which can be attributed to their abundant phenolic hydroxyl groups. Their activity in mitigating indirect oxidative damage is unmatched within this class of compounds. However, the clinical translation of polyphenols is significantly hindered by poor bioavailability; they undergo rapid metabolic inactivation following oral administration, and high doses may induce pro-oxidative effects. Critically, they do not provide protection against direct DNA damage caused by physical radiation interactions. Consequently, their use as single-agent radioprotectants is limited, necessitating the development of advanced delivery systems to enhance therapeutic efficacy. The protective effects of polyphenols against radiation-induced damage are shown in Table 1.

**TABLE 1:** Protective effect of polyphenolic substances on radiation-induced damage.

Active compound	Dose	Radiation dose	Model	Mechanism	References
5,7-DHF	25 mg/kg	5 Gy,^137^Cs-γ	Wister rat	Downregulation of caspase-3 activity	Mansour et al. (2017)
Curcumin	100 mg/kg	6 Gy, X-ray	Wistar rat	Inhibition of NF-κB signaling pathway	Azmoonfar et al. (2025)
RES	20 mg/kg	8 Gy,^137^Cs-γ	Wistar rat	Reduced TNF-α, IL-1β, and IFN-γ levels; decreased expression of Bax and caspase-3 proteins	Ali and Radwan (2021)
Hesperidin	10 μM	2 Gy, X-ray	Caco-2 cell	Activation of CaMKK/AMPK pathway to modulate tight junction proteins (occludin, claudin-4)	Park and Yu (2024)
PT and SIL	PT (100 mg/kg) SIL (70 mg/kg)	7.2 Gy,^137^Cs-γ	Swiss albino mice	Downregulation of mutant p53 protein; upregulation of Bax expression	Obrador et al. (2023)
EGCG	25 mg/kg	9 Gy,^60^Co-γ	C57BL/6 mice	Upregulate the expression of HO-1, GPX4, and SLC7A11	Xie et al. (2020)
TFERL	40 μg/mL	7.2 Gy,^137^Cs-γ	C57BL/6J mice	Reduce the levels of TNF-α and IL-6, and enhance the activity of SOD and GSH	Wu et al. (2023)
NAR	100 mg/kg	13 Gy, X-ray	C57BL/6J mice	Reduce the secretion of TNF-α, IL-1β, and IL-6	Ling et al. (2024)
Q-3-R	10 mg/kg	7.5 Gy,^60^Co-γ	C57BL/6 mice	Differential Regulation of Bcl-2, Bax, and NF-κB Associated with Reduced Serum TNF-α Levels	Dutta et al. (2021)
UroA	2 mg/kg	9 Gy,^137^Cs-γ	C57BL/6 mice	Inhibition of p53-mediated apoptosis is associated with gut microbiota remodeling	Zhang et al. (2021)

### 3.2 Polysaccharides

Polysaccharides are high-molecular-weight polymers formed by the linkage of monosaccharides via glycosidic bonds and are abundantly found in plants, fungi, and marine organisms. Their structural diversity confers unique biological activities. Research has demonstrated that polysaccharides exhibit significant biological activities, which include anti-tumor, antioxidant, anti-diabetic, anti-radiation, lipid-lowering, and immunomodulatory effects (Zeng et al., 2019).

Astragalus polysaccharides (APS), the principal active component of the traditional Chinese medicine Astragalus, have demonstrated potential preventive and therapeutic effects against radiation-induced heart disease (RIHD). Experimental data indicate that APS intervention significantly enhances cardiac function indices in rat models of radiation-induced heart disease (RIHD), mitigates pathological damage to myocardial tissue, and reduces serum levels of inflammatory factors. Further mechanistic studies suggest that the cardioprotective effects of APS may be mediated by the regulation of the PI3K/Akt/mTOR signaling pathway, which is essential for maintaining cardiomyocyte survival, regulating autophagy, and inhibiting apoptosis (Jiang et al., 2025). Lycium barbarum polysaccharides (LBP), the primary active component of the medicinal plant Lycium barbarum L. from the Solanaceae family, exhibit significant biological activities. Research has demonstrated that LBP effectively protects the reproductive function of male rats exposed to low-dose localized radiation by upregulating the expression of the anti-apoptotic protein Bcl-2, downregulating the expression of the pro-apoptotic protein Bax, and enhancing mitochondrial membrane potential (MMP) (Luo et al., 2014). Ginseng polysaccharide GPS-2 (GPS-2) is a polysaccharide component with notable biological activity derived from the medicinal plant ginseng. Feng’s team discovered that GPS-2 can alleviate the inflammatory response induced by 6 Gy of ^60^Co-γ irradiation in mice, achieving its protective effect by promoting the secretion of IFN-γ and interleukin-4 (IL-4) (Feng et al., 2024). Schisandra polysaccharide (SP), a bioactive polysaccharide component extracted from Schisandra chinensis (SC), has been shown by Zhao’s research group to mitigate radiation-induced thymocyte damage by modulating the expression of apoptosis-associated proteins. Mechanistic studies indicate that SP exerts its anti-apoptotic effects by significantly upregulating the expression of Bcl-2, an anti-apoptotic protein, while simultaneously downregulating the expression levels of the pro-apoptotic proteins Fas and Bax in irradiated thymocytes (Zhao et al., 2015). Beta-D-Glucan extracted from *Saccharomyces cerevisiae* has demonstrated significant radioprotective properties. Research indicates that Beta-D-Glucan can reduce intracellular levels of ROS and malondialdehyde (MDA), thereby mitigating radiation-induced DNA damage and cellular apoptosis. It further enhances cell viability by modulating the cell cycle and mitigating radiation-induced cell cycle arrest It further enhances cell viability by modulating the cell cycle and mitigating radiation-induced cell cycle arrest. This polysaccharide also activates the NF-κB signaling pathway within cells, upregulating the activity of antioxidant enzymes and enhancing cellular antioxidant capacity (Liu et al., 2019). Annona squamosa leaf polysaccharides (ASLP) are a class of naturally occurring active polysaccharides extracted from the leaves of Annona squamosa L., which exhibit significant biological activities. Their chemical composition primarily consists of glucose, galactose, and arabinose, often including acidic polysaccharide components. Research conducted by Byun’s team has demonstrated that ASLP can protect normal human epidermal keratinocytes and mouse skin from radiation damage. This protective effect is achieved by reducing the levels of IL-1β, nucleotide-binding domain and leucine-rich repeat-containing family pyrin 3 (NLRP3), as well as the cleavage of caspase-1 and caspase-3 (Byun et al., 2020). Sipunculus nudus L., an edible marine organism, represents a promising source of functional seafood owing to its significant bioactivities, which include anti-hypoxic, anti-fatigue, immunomodulatory, antibacterial, and antioxidant properties. The bioactive component Sipunculus nudus Linnaeus polysaccharide (SNP) exhibits substantial radioprotective effects. In radiation-injured animal models, administration of SNP not only prolongs survival but also significantly enhances SOD and GSH-PX activities while decreasing MDA levels (Li et al., 2016). The acidic heteropolysaccharide Dicliptera chinensis polysaccharide (DCP), derived from the plant Dicliptera chinensis of the Acanthaceae family, primarily consists of glucose, galactose, and arabinose. Huang et al. have demonstrated that DCP exerts anti-radiation fibrosis effects by inhibiting the activation of the Transforming Growth Factor beta 1/Small Mothers Against Decapentaplegic/Connective Tissue Growth Factor (TGF-β1/Smads/CTGF) signaling pathway (Huang et al., 2025). Neutral polysaccharides from Hohenbuehelia serotina (NTHSP) are the active polysaccharide components derived from the edible fungus Hohenbuehelia serotina. Research conducted by Wang’s team has demonstrated that NTHSP effectively inhibits the endoplasmic reticulum-mitochondrial apoptosis cascade induced by γ-ray radiation. This inhibition occurs through the specific regulation of the ER stress-mediated apoptotic signaling network, which encompasses three core pathways: PERK-ATF4-CHOP, IRE1α-XBP1-CHOP, and ATF6-XBP1-CHOP. This significantly decreases the apoptosis rate of splenic lymphocytes (Wang and Li, 2019).

Polysaccharides demonstrate the most significant efficacy in alleviating radiation-induced bone marrow suppression and immune exhaustion. This activity is mediated by the activation of immune cells and the promotion of hematopoietic stem cell regeneration, which constitutes a key mechanism for enhancing survival rates in acute radiation syndrome. Due to their large molecular weight, which results in poor oral absorption, polysaccharides primarily exert their radioprotective effects indirectly through immune regulation. The protective effects of polysaccharides against radiation-induced damage are shown in Table 2.

**TABLE 2:** Protective effect of polysaccharides on radiation-induced damage.

Active compound	Dose	Radiation dose	Model	Mechanism	References
APS	200,400,800 mg/kg	40 Gy, X-ray	Wister rat H9C2 cell	Modulation of PI3K/Akt/mTOR signaling pathway	Jiang et al. (2025)
LBP	10 mg/kg	2.3 Gy,^60^Co-γ	Wistar rat	Upregulation of Bcl-2 expression; downregulation of Bax expression	Luo et al. (2014)
GPS-2	50, 100, 200 mg/kg	6 Gy,^60^Co-γ	C57BL/6 mice BALB/c mice	Promotion of IFN-γ and IL-4 secretion	Feng et al. (2024)
SP	4 g/kg	6 Gy,^60^Co-γ	BALB/c mice	Increased Bcl-2 expression; decreased Fas and Bax levels	Zhao et al. (2015)
Beta-D-Glucan	350 mg/kg	6 Gy,^12^C^6+^-ray	BALB/c mice	Activation of NF-κB signaling pathway	Liu et al. (2019)
ASLP	50 μg/mL	7 Gy,^137^Cs-γ	NHEK cell	Reduction of IL-1β and NLRP3 levels; cleavage of caspase-1 and caspase-3	Byun et al. (2020)
SNP	30, 270 mg/kg	4 Gy,^137^Cs-γ	BALB/c mice	Enhances SOD and GSH-PX activities while reducing MDA levels	Li et al. (2016)
DCP	200 μg/mL	8 Gy, X-ray	Rat Dermal Fibroblasts	Decreased the expression of α-SMA, TGF-β 1, Smad3 and CTGF	Huang et al. (2025)
NTHSP	200 mg/kg	6 Gy,^60^Co-γ	KM mice	The ER apoptosis pathways mediated by PERK-ATF4-CHOP, IRE1α-XBP1-CHOP, and ATF6-XBP1-CHOP.	Wang and Li (2019)

### 3.3 Saponins

Saponins are a class of glycoside compounds that are widely found in natural products, primarily distributed in terrestrial higher plants such as ginseng, soybeans, and astragalus. They are also present in smaller amounts in marine organisms, including starfish and sea cucumbers. Due to their structural diversity and broad biological activities, these compounds have demonstrated significant potential for application in various fields, including antibacterial, antipyretic, sedative, and anticancer effects. Consequently, they are gradually emerging as a key focus in natural product research. As the primary active component in ginseng, Ginsenoside Rg3 (GRg3) has been shown to inhibit the excessive release of pro-inflammatory factors by modulating the TLR4/Myeloid Differentiation Primary Response 88 (MyD88)/NF-κB pathway. Additionally, it reshapes the balance of the gut microbiota by significantly increasing the abundance of beneficial bacteria and reducing inflammatory responses (Duan et al., 2022). This finding confirms that GRg3 has the potential to serve as a novel therapeutic target for acute radiation proctopathy (ARP), offering a complementary strategy to conventional supportive therapies. Panax notoginseng saponins (PNS), the primary active components derived from the medicinal plant Panax notoginseng of the Araliaceae family, belong to tetracyclic triterpenoid saponins and exhibit a diverse array of pharmacological effects. Research has indicated that PNS not only exhibit fundamental physiological regulatory functions, including antithrombotic, neuroprotective, and cardioprotective effects, but also demonstrate potential applications in anti-tumor activity, oxidative stress regulation, intestinal microecological balance, and regulation of bone metabolism (Bo et al., 2020). Du’s team has experimentally confirmed that Panax notoginseng saponins (PNS) exhibit significant preventive and therapeutic effects against radiation-induced osteoporosis. Its mechanism of action involves the bidirectional regulation of bone tissue remodeling: promoting bone formation-related signaling pathways on one hand, and inhibiting osteoclast-mediated bone resorption on the other. The study further reveals that this component can exert protective effects on bone by modulating the balance of the inflammatory factor network. Specifically, it reduces the expression levels of pro-inflammatory factors such as IFN-γ, TNF-α, IL-6, and Interleukin-17 (IL-17), while enhancing the biological activity of the anti-inflammatory factor IL-10. This modulation effectively ameliorates radiation-induced bone metabolic disorders (Wenxi et al., 2015). Asterosaponin P1 is a steroidal saponin compound isolated from marine echinoderms, specifically starfish, and represents a unique secondary metabolite within these organisms. Recent research has demonstrated that Asterosaponin P1 exhibits radiosensitizing activity, effectively reducing both the number and size of colonies in human colorectal cancer cells. This effect is mediated by modulating the expression of the anti-apoptotic protein B-cell lymphoma Extra Large (Bcl-XL) and the pro-apoptotic protein Bax, ultimately resulting in the programmed cell death of tumor cells via the cascade activation of the caspase-9/caspase-3 protease system (Malyarenko et al., 2019). Saikosaponin A (SSA), the principal active compound isolated from the roots of the traditional Chinese herb Bupleurum chinense DC, is classified as a pentacyclic triterpenoid saponin. The research team led by Kim identified that it can mitigate drug resistance in gastric cancer cells during radiation therapy by targeting ER stress. This effect is mediated by inducing G0/G1 cell cycle arrest in SMMC-7721 hepatocellular carcinoma cells, upregulating the expression of p53 and Bax, and downregulating the expression of Bcl-2 in response to hypoxic conditions, thereby exerting a potent anticancer effect (Kim, 2023). Ecliptae Herba (EH), a member of the Asteraceae family, is traditionally recognized for its medicinal properties, including hemostasis and the promotion of blood circulation to alleviate blood stasis. Widely distributed in tropical and subtropical regions, it holds significant value in ethnomedicine. Ecl2 is the main component of triterpenoid saponins in Ecliptae Herba. Current research has confirmed the efficacy of Ecl2 in ameliorating radiation-induced enteritis by inhibiting IR-triggered endothelial cell apoptosis through the mechanistic activation of PPARγ, which subsequently suppresses the Bax/Bcl-2-Caspase-3/PARP signaling cascade (Zeng et al., 2025). Korean Red Ginseng Saponins (RGSF) are the primary active components derived from the steamed processing of Panax ginseng C.A. Meyer, a specialty variety of red ginseng from the Korean Peninsula. Current research indicates that RGSF can synergistically inhibit the DNA damage response and inflammatory pathways by blocking Chk2 phosphorylation, inhibiting NF-κB nuclear translocation, and downregulating HO-1 expression. This action significantly reduces the burst generation of NO induced by the combined effects of IR and lipopolysaccharide (LPS) (Lee et al., 2014).

Saponins effectively inhibit radiation-induced apoptosis by modulating pathways such as PI3K/Akt, demonstrating superior protective efficacy for sensitive tissues, including the intestinal epithelium and germ cells, compared to other compound classes. However, the application of saponins faces challenges related to both safety and complexity. Hemolytic toxicity restricts the available administration routes, while significant variations in activity among individual saponins hinder standardized implementation. The protective effects of saponins against radiation-induced damage are shown in Table 3.

**TABLE 3:** Protective effect of saponins on radiation-induced damage.

Active compound	Dose	Radiation dose	Model	Mechanism	References
GRg3	1600 μg/mL	6 Gy,^60^Co-γ	C57BL/6 mice BALB/c mice	Modulation of TLR4/MyD88/NF-κB pathway; inhibition of pro-inflammatory cytokine overproduction	Duan et al. (2022)
PNS	100 mg/kg	8 Gy,^60^Co-γ	BALB/c mice	Downregulation of IFN-γ, TNF-α, IL-6, and IL-17; enhancement of IL-10 bioactivity	Wenxi et al. (2015)
Asterosaponin P1	150 μM	2 Gy, X-ray	DLD-1、HCT 116 and HT-29 cell	Regulation of Bcl-XL/Bax ratio; activation of caspase-9/caspase-3 cascade	Malyarenko et al. (2019)
SSA	10 μM	6 Gy,^137^Cs-γ	Human GC cell lines	Regulation of the Bcl-XL/Bax ratio and the activation of the caspase-9/caspase-3 cascade	Kim (2023)
Ecl2	50 mg/kg	13 Gy, X-ray	C57BL/6 mice	Suppressing the Bax/Bcl-2-Caspase-3/PARP signaling cascade	Zeng et al. (2025)
RGSF	10 μg/mL	10 Gy,^137^Cs-γ	RAW264.7 cell	Inhibit the CHK2, NF-κB, and HO-1 signaling pathways	Lee et al. (2014)

### 3.4 Alkaloids

Alkaloids are a class of nitrogen-containing organic compounds predominantly derived from higher plants, particularly dicotyledons. They are found in plants in various forms, including free bases, salts, glycosides, and amides. Alkaloids occupy a significant position in pharmacology and medicine due to their diverse biological activities, including antibacterial, antitumor, anti-inflammatory, and antioxidant properties.

Matrine, a quinolizidine alkaloid derived from the roots of the leguminous plant Sophora flavescens Ait., has been demonstrated to alleviate cellular damage in functional hematopoietic stem cells (HSCs) following irradiation with 300 cGy of ^60^Co-γ rays.

This effect is mediated by the activation of the MAPK signaling pathway, which upregulates the expression of downstream effectors, thereby enhancing HSC proliferation and decreasing IR-induced apoptosis (Yang et al., 2023). Piperine (PIP) is an amide alkaloid derived from the fruits or rhizomes of Piper nigrum L. and Piper longum L. It has been traditionally utilized for the regulation of digestive system functions and the treatment of respiratory diseases. The research conducted by Diehl et al. has revealed the potential value of this compound in the field of tumor radiotherapy. Specifically, it can significantly enhance the sensitivity of melanoma cells to IR by regulating the mitochondrial-dependent apoptotic pathway, thereby exerting a radiosensitizing effect. This effect is accomplished through the selective inhibition of the anti-apoptotic protein Bcl-2 expression, while significantly upregulating the activity levels of the pro-apoptotic protein Bax and the apoptotic execution factor caspase-9, thereby promoting apoptosis (Diehl et al., 2022). Furthermore, research conducted by Safarbalou et al. has demonstrated that PIP can provide protection against radiation-induced colon injury. Pretreatment of irradiated mice with PIP effectively mitigates oxidative stress in colon tissues, as indicated by decreased levels of MDA and protein carbonyl (PC), while preserving GSH content following IR exposure (Safarbalou et al., 2024). Biscoclaurine alkaloids (BA) are extracted from the traditional medicinal plant Angelica dahurica, which has a longstanding history of use in clinical practice within traditional Chinese medicine. It is commonly utilized to treat conditions such as mumps, gastric ulcers, and leukopenia. Research indicates that BA can alleviate insulin resistance-induced leukopenia and thrombocytopenia by increasing white blood cell and platelet counts, enhancing immune function, and upregulating antioxidant capacity. This protective effect is mediated by the activation of the Nrf2/Antioxidant Response Element (ARE) pathway, which enhances the activity of endogenous antioxidant enzymes, including SOD and catalase (CAT), while also increasing the levels of vascular cell adhesion molecule 1 (VCAM-1) and IFN-γ (Wang et al., 2020). Tetramethylpyrazine (Ligustrazine), the principal active component of Ligusticum chuanxiong Hort., has been demonstrated by Zheng et al. through systematic experiments to provide protection against γ-radiation damage. This compound specifically inhibits the activation of the p38 mitogen-activated protein kinase (p38 MAPK) signaling pathway induced by radiation, significantly downregulates the secretion of pro-inflammatory cytokines TNF-α and IL-6 following radiation exposure, and is closely associated with its regulation of NF-κB nuclear translocation and inhibition of NLRP3 inflammasome activation (Zheng et al., 2013). Berberine (BBR), a prominent component of benzylisoquinoline alkaloids, is extensively found in medicinal plants belonging to the Berberidaceae and Papaveraceae families. Recent studies have demonstrated that BBR can enhance the radiosensitivity of liver cancer by modulating the cell cycle and senescence pathways. This effect is achieved through the activation of the ATM-Chk1 pathway and the promotion of p21 overexpression, resulting in G2/M phase arrest, while simultaneously increasing the level of the senescence-associated cytokine IL-6 (Ramesh et al., 2020). Furthermore, Tu’s team has confirmed through clinical trials that BBR can significantly alleviate gastrointestinal symptoms in patients with radiation-induced enteritis. Mechanistic studies have revealed that BBR effectively enhances the regenerative capacity of intestinal stem cells (ISCs) in both healthy and radiation-damaged mouse models by synergistically activating the mammalian target of rapamycin complex 1 (mTORC1), signal transducer and activator of transcription 3 (STAT3), and extracellular signal-regulated kinase 1/2 (ERK1/2) signaling pathways (Tu et al., 2024). Orychophragine D (1) was isolated from the seeds of *Orychophragmus violaceus*. Studies have shown that it can enhance levels of white blood cells (WBC), red blood cells (RBC), platelets (PLT), and hemoglobin (HGB) in mice, improve survival rates following exposure to ionizing radiation, and facilitate the recovery of the hematopoietic system after total body irradiation (TBI) (Zhang et al., 2023).

Alkaloids exhibit preferential efficacy in suppressing radiation-induced inflammatory cascades, particularly through the inhibition of the NF-κB pathway. This activity is primarily mediated through the blockade of pro-inflammatory cytokine production and the downregulation of cyclooxygenase-2, which constitutes a key mechanism for attenuating secondary tissue damage. Alkaloids exert limited radioprotective effects and may paradoxically enhance radiosensitivity in certain structural contexts due to their narrow therapeutic indices and inherent compound-specific toxicity risks. The protective effects of alkaloid substances against radiation-induced damage are shown in Table 4.

**TABLE 4:** Protective effect of alkaloids on radiation-induced damage.

Active compound	Dose	Radiation dose	Model	Mechanism	References
Matrine	10 mg/kg	500, 750 cGy^60^Co-γ	C57BL/6 mice	Activation of MAPK signaling pathway; upregulation of Cyclin D1 and Bcl-2	Yang et al. (2023)
PIP	100, 200 μM	6 Gy, X-rays	T98G cell FaDu cell	Inhibition of Bcl-2; upregulation of Bax and caspase-9 activity	Diehl et al. (2022)
PIP	10 mg/kg	6 Gy, X-rays	BALB/c mice	Reduce MDA and PC levels, maintain GSH content	Safarbalou et al. (2024)
BA	10,20 mg/kg	250 cGy,^60^Co-γ	Kunming mice	Activation of Nrf2/ARE pathway; enhanced SOD and CAT activity; increased VCAM-1 and IFN-γ levels	Wang et al. (2020)
TMP	20,40 mg/kg	9.5 Gy,^60^Co-γ	C57BL/6 mice	Downregulation of TNF-α and IL-6 secretion	Zheng et al. (2013)
BBR	100 μM	8 Gy, X-ray	HepG2 cell	Activation of ATM-Chk1 pathway; p21 overexpression; elevated IL-6 levels	Ramesh et al. (2020)
BBR	5 mg/kg	8 Gy X-ray	C57BL/6 mice	Activation of the mTORC1, STAT3, and ERK1/2 signaling pathways	Tu et al. (2024)
Orychophragine D (1)	50 mg/kg	8 Gy,^60^Co-γ	SPF C57BL mice	Significantly enhance WBC, RBC, PLT, and HGB	Zhang et al. (2023)

### 3.5 Polypeptides

Natural product peptides are bioactive molecules formed by the linkage of amino acids through peptide bonds and are widely found in plants, animals, and microorganisms. Their structural diversity endows them with various biological activities, including antioxidant, anti-inflammatory, and immune-regulatory effects, particularly excelling in the area of radiation protection.

*Tenebrio molitor* is a novel edible insect species that serves as a rich source of high-protein food. Research indicates that peptides derived from *T. molitor* (TMP) exhibit beneficial effects on restoring hematopoietic system function and preserving intestinal integrity. TMP significantly reduces the expression levels of pro-inflammatory factors TNF-α, IL-1β, and IL-6 by inhibiting signaling pathways such as NF-κB, while simultaneously upregulating the synthesis of tight junction proteins Zonula Occludens-1 (ZO-1) and Occludin, thereby maintaining the structural integrity of the intestinal epithelium (Shang et al., 2024). Ginseng oligopeptides (GOP) are a class of natural active substances characterized by low molecular weight and high bioavailability, and they exhibit numerous potential physiological functions. Researchers have found that it effectively alleviates intestinal damage and reduces oxidative stress and inflammatory responses induced by radiation. The molecular mechanism underlying its anti-inflammatory effects is primarily mediated by the inhibition of the NF-κB signaling pathway and a reduction in the production of TNF-α (He et al., 2017). Vasoactive intestinal peptide (VIP) is a neuropeptide that is widely distributed throughout both the central and peripheral nervous systems. It serves not only as an important neuromodulator and neurotransmitter but also as a natural polypeptide with extensive physiological effects. Current research indicates that vasoactive intestinal peptide (VIP) can further enhance cellular tolerance to radiation damage by inducing the activation of the p53 pathway, thereby reducing apoptosis and necrosis in cells. Simultaneously, VIP can significantly decrease the production of the inflammatory cytokine TNF-α, thereby inhibiting the excessive activation of subsequent inflammatory responses (Agibalova et al., 2024). SS31 is a small molecular peptide composed of four amino acids, distinguished by its significant mitochondrial targeting properties. Its primary mechanism of action involves targeting the inner mitochondrial membrane and interacting with cardiolipin, thereby safeguarding the structure and function of mitochondria (Tung et al., 2025). Research has demonstrated that SS31 can significantly mitigate radiation-induced mitochondrial damage, thereby effectively preventing injury to the hematopoietic system caused by IR. SS31 not only scavenges superoxide radicals in mitochondria but also eliminates ROS and superoxide radicals derived from hydrogen peroxide (H_2_O_2_) in lineage-specified progenitor (LSK) cells, hematopoietic progenitor cells (HPC), hematopoietic stem cells (HSC), and multipotent progenitor cells (MPP) (Han et al., 2019). Therefore, SS31 can prevent IR-induced damage to the hematopoietic system by clearing various types of ROS. This finding provides an important theoretical foundation for the application of SS31 in the field of radiation protection. Walnut oligopeptides (WOP) are a mixture of small molecular peptides that have been extracted and isolated from walnut protein. Zhu’s team discovered that it improves acute injuries in mice caused by IR and accelerates their recovery. This effect is associated with its inherent antioxidant activity, protection of immune organs and the intestinal barrier, and inhibition of cellular apoptosis. It reduces oxidative damage by upregulating the tight junction proteins occludin and ZO-1, while simultaneously suppressing the activation of NF-κB to diminish the production of IL-1β, IL-6, and TNF-α (Zhu et al., 2019). Whey hydrolysate peptides (WHP) are a functional mixture of short peptides derived from the enzymatic hydrolysis of whey protein. Research indicates that WHP can significantly extend the survival time of irradiated mice, promote body weight recovery, increase the number of white blood cells in peripheral blood, and enhance DNA content in bone marrow. Its protective mechanism involves inhibiting the activation of the NF-κB pathway, downregulating the expression of pro-inflammatory factors such as TNF-α and IL-6, while simultaneously increasing the activity of SOD and glutathione peroxidase 4 (GPx1) in serum and liver, and significantly reducing the levels of MDA in both serum and liver (Liu et al., 2021). Glucagon-like peptide-1 (GLP-1) and glucagon-like peptide-2 (GLP-2) are synthesized and secreted by intestinal L-cells as post-translational cleavage products of the proglucagon gene. Research has shown that both peptides mitigate radiation-induced oxidative damage in the ileum and colon. Mechanistically, GLP-1 reduces IR-induced injury concomitantly with decreased MDA levels in the ileum, whereas GLP-2 treatment promotes GSH replenishment in rats (Deniz et al., 2015). Ghrelin is a polypeptide hormone that is naturally secreted by the gastrointestinal tract of mammals, predominantly synthesized by P/D1 cells located in the gastric fundus. Research conducted by Kiang et al. has demonstrated that ghrelin can mitigate intestinal damage resulting from the combined effects of radiation and skin trauma. Mechanistic research has indicated that ghrelin treatment enhances the phosphorylation activation of the AKT and ERK signaling pathways, inhibits the phosphorylation activation of Jun N-terminal Kinase (JNK) and the activation of caspase-3 in ileal tissue, and reduces the expression levels of NF-κB, inducible nitric oxide synthase (iNOS), and BAX (Kiang et al., 2020).

Peptides exhibit significant efficacy in mitigating radiation-induced oxidative stress through the rapid scavenging of ROS, particularly hydroxyl radicals. This activity primarily occurs through the direct donation of electrons from functional groups and the enzymatic decomposition of superoxides, representing a fundamental defense mechanism against macromolecular damage. However, susceptibility to proteolytic degradation and limited membrane permeability restrict their action to intracellular antioxidant pathways, thereby precluding the systemic immunomodulatory effects observed in other radioprotectants. The protective effects of polypeptide substances against radiation-induced damage are presented in Table 5.

**TABLE 5:** Protective effect of peptide substances on radiation-induced damage.

Active compound	Dose	Radiation dose	Model	Mechanism	References
TMP	0.6 g/kg	3 Gy, infrared radiation	BALB/c mice	Inhibition of NF-κB signaling; reduced TNF-α, IL-1β, and IL-6 expression; upregulation of ZO-1 and occludin synthesis	Shang et al. (2024)
GOP	100 g/mL	8 Gy,^60^Co-γ	BALB/c mice	Suppression of NF-κB pathway; decreased TNF-α production	He et al. (2017), Agibalova et al. (2024)
VIP	750 g/kg	12 Gy, X-ray	C57Bl6/J mice	Reduction of TNF-α; inhibition of hyperactivated inflammatory cascades	Tung et al. (2025)
SS31	6 mg/kg	4 Gy, infrared radiation	C57BL/6 mice ICR mice	Scavenging ROS to prevent irradiation-induced hematopoietic damage	Han et al. (2019)
WOP	0.44, 0.88 g/kg	8 Gy,^60^Co-γ	BALB/c mice	Inhibition of NF-κB activation; reduced IL-1β, IL-6, and TNF-α production	Zhu et al. (2019)
WHP	0.3 g/kg	8 Gy,^60^Co-γ	BALB/c mice	Downregulate the expression of pro-inflammatory factors TNF-α and IL-6, while increasing the activity of SOD and GPx1	Liu et al. (2021)
GLP-1 and GLP-2	0.1 nmol/kg; 7 nmol/kg	11 Gy, X-ray	Sprague-Dawley rat	Reduce the MDA level in the ileum and promote the replenishment of glutathione GSH	Deniz et al. (2015)
Ghrelin	113 μg/kg	9.5 Gy,^60^Co-γ	B6D2F1/J mice	Alleviated the increase of IL-1β, IL-6, IL-17A, IL-18, and TNF-α in serum	Kiang et al. (2020)

## 4 Conclusion

The pathogenesis of tissue damage induced by ionizing radiation is rooted in oxidative stress cascades triggered by the burst of free radicals, which include DNA double-strand breaks, mitochondrial dysfunction, and hyperactivation of inflammatory signaling pathways. These processes ultimately result in immunosuppression, hematopoietic failure, and multi-organ damage. Confronting this complex pathological network, conventional synthetic radioprotectors, such as amifostine and corticosteroids, offer only transient symptom relief while posing significant toxicity and immunosuppressive risks. In contrast, natural compounds with established radioprotective potential—such as polyphenols, polysaccharides, saponins, alkaloids, and peptides—demonstrate unique advantages via multi-target synergistic mechanisms. The key mechanisms include potent free radical scavenging, enhanced DNA repair, mitochondrial protection, and modulation of inflammatory pathways, including NF-κB. Collectively, these actions maintain homeostasis in the host microenvironment while significantly reducing hepatorenal toxicity, thereby paving the way for the development of next-generation radioprotectants.

Despite their potential, the clinical translation of these bioactive components encounters significant challenges, including inefficient screening of core active constituents, undefined *in vivo* metabolic pathways, and inadequate analysis of multi-target interaction networks. Importantly, distinct compound classes exhibit varying pharmacokinetic profiles. Lipophilic alkaloids tend to accumulate in specific organs, resulting in dose-dependent toxicity, whereas hydrophilic polysaccharides demonstrate low bioavailability due to absorption barriers. These inherent limitations directly hinder the translation of the protective efficacy observed in animal models to clinical applications. Future breakthroughs should concentrate on three key areas: 1) the comprehensive integration of metabolomics, single-cell sequencing, and deep learning technologies to accurately analyze the structure-activity relationships of active molecules and their cross-organ regulatory networks; 2) the optimization of compound bias design strategies, such as enhancing polysaccharide delivery efficiency using nanocarriers or minimizing the accumulation toxicity of lipophilic components through structural modifications, thereby systematically improving bioavailability and *in vivo* stability; and 3) the establishment of a preclinical translational evaluation system focused on validating the applicability of multi-component synergistic effects within the human microenvironment. With the comprehensive elucidation of the mechanisms underlying natural products and the advancement of synthetic biology technologies, radiation protection strategies that leverage the multi-target synergy and low toxicity of natural compounds are anticipated to address precision bias bottlenecks, facilitate the introduction of new products such as nanoformulations into clinical applications, and ultimately deliver highly efficient and safe solutions for nuclear protection and aerospace medicine.
